# Subependymal Giant Cell Astrocytoma Mimicking Recurrent Ependymoma: Imaging Clues to the Diagnosis of Tuberous Sclerosis Complex

**DOI:** 10.7759/cureus.117140

**Published:** 2026-09-29

**Authors:** Abdelilah Bouymajjan, Asmae Oulad Amar, El Mahdi Choukri, Ali Meqor, Siham Alaoui Rachidi

**Affiliations:** 1 Diagnostic and Interventional Radiology, Mohammed VI University Hospital, Tangier, MAR; 2 Faculty of Medicine and Pharmacy, Abdelmalek Essaâdi University, Tangier, MAR

**Keywords:** chronic calcified subdural hematomas, intracranial ependymoma, obstructive hydrocephalus, subependymal giant cell astrocytoma (sega), tuberous sclerosis complex (tsc)

## Abstract

Tuberous sclerosis complex (TSC), also known as Bourneville disease, is a multisystem genetic disorder that can involve the central nervous system. We report the case of an adolescent with a history of intraventricular ependymoma resection during childhood and ventriculoperitoneal shunt placement who presented with symptoms of intracranial hypertension. Brain imaging revealed a right intraventricular solid-cystic mass involving the foramen of Monro, associated with subependymal nodules and cortical tubers, suggesting previously undiagnosed TSC. This case emphasizes the importance of recognizing imaging features of TSC in patients presenting with intraventricular tumors to ensure accurate diagnosis and appropriate management.

## Introduction

Tuberous sclerosis complex (TSC) is an autosomal dominant genetic disorder caused by mutations in the TSC1 or TSC2 genes, leading to constitutive activation of the mammalian target of rapamycin (mTOR) signaling pathway. It is a multisystem disorder characterized by hamartomas, benign tumor-like growths composed of mature but disorganized tissues, affecting the brain, kidneys, heart, and skin. Neurological manifestations include cortical tubers and subependymal nodules, while renal angiomyolipomas, cardiac rhabdomyomas, and hypomelanotic macules are among other characteristic findings [1].

According to the 2021 WHO classification, subependymal giant cell astrocytoma (SEGA) is a WHO grade 1 circumscribed astrocytic glioma strongly associated with TSC [2]. It typically arises at the caudothalamic groove near the foramen of Monro and may cause obstructive hydrocephalus. A SEGA is defined radiologically by a subependymal lesion at the caudothalamic groove measuring more than one cm or by documented serial growth [3]. Regular MRI surveillance is recommended in patients with TSC. Management may include surgical resection or mTOR inhibitors, which target the dysregulated mTOR pathway underlying TSC and have been shown to reduce SEGA growth, providing an effective alternative to surgery in selected patients [1,3].

In adolescents presenting with an intraventricular tumor, ependymoma may represent an important differential diagnosis, particularly because ependymomas can occur in the pediatric and adolescent population and may present with obstructive hydrocephalus. However, their typical locations and imaging characteristics differ from those of a lesion centred at the caudothalamic groove or foramen of Monro. Central neurocytoma may also be considered in the differential diagnosis, although it typically occurs in young adults, most commonly before the age of 40 years [4]. This case highlights the diagnostic challenge of distinguishing SEGA from recurrent ependymoma and emphasizes the importance of recognizing previously unrecognized manifestations of TSC.

## Case presentation

We report the case of an 18-year-old male with no known family history of tuberous sclerosis complex (TSC), who underwent surgical resection of an intraventricular ependymoma at the age of four years, with placement of a ventriculoperitoneal shunt. According to his parents, there were no subsequent hospitalizations, additional treatments, or shunt revisions during the intervening years. There was also no reported history of seizures, developmental or cognitive impairment, or other known TSC-related manifestations. The patient presented with a one-month history of symptoms of intracranial hypertension, including headaches, vomiting, blurred vision, and progressive gait instability. Neurological examination revealed static and kinetic cerebellar ataxia, decreased visual acuity, and bilateral optic disc pallor. Cutaneous examination revealed five hypomelanotic macules, all measuring more than 5 mm, with the largest measuring 14 mm, distributed over the back and upper limbs. Laboratory test results were unremarkable. 

Non-contrast and contrast-enhanced brain CT demonstrated a right intraventricular mass centred at the foramen of Monro and extending into the septum pellucidum. The lesion exhibited mixed solid and cystic components, peripheral calcifications, and heterogeneous enhancement associated with tetraventricular hydrocephalus and transependymal cerebrospinal fluid resorption. Additional findings included multiple calcified subependymal and intraparenchymal nodules, bilateral chronic calcified subdural hematomas, and an in situ ventriculoperitoneal shunt (Figure 1). Cortical tubers were not identified on the initial CT, which has lower sensitivity than MRI for their detection. Urgent ventriculoperitoneal shunt revision was performed, resulting in clinical improvement. Intraoperative cerebrospinal fluid analysis demonstrated elevated protein levels with normal glucose concentration.

**Figure 1:**
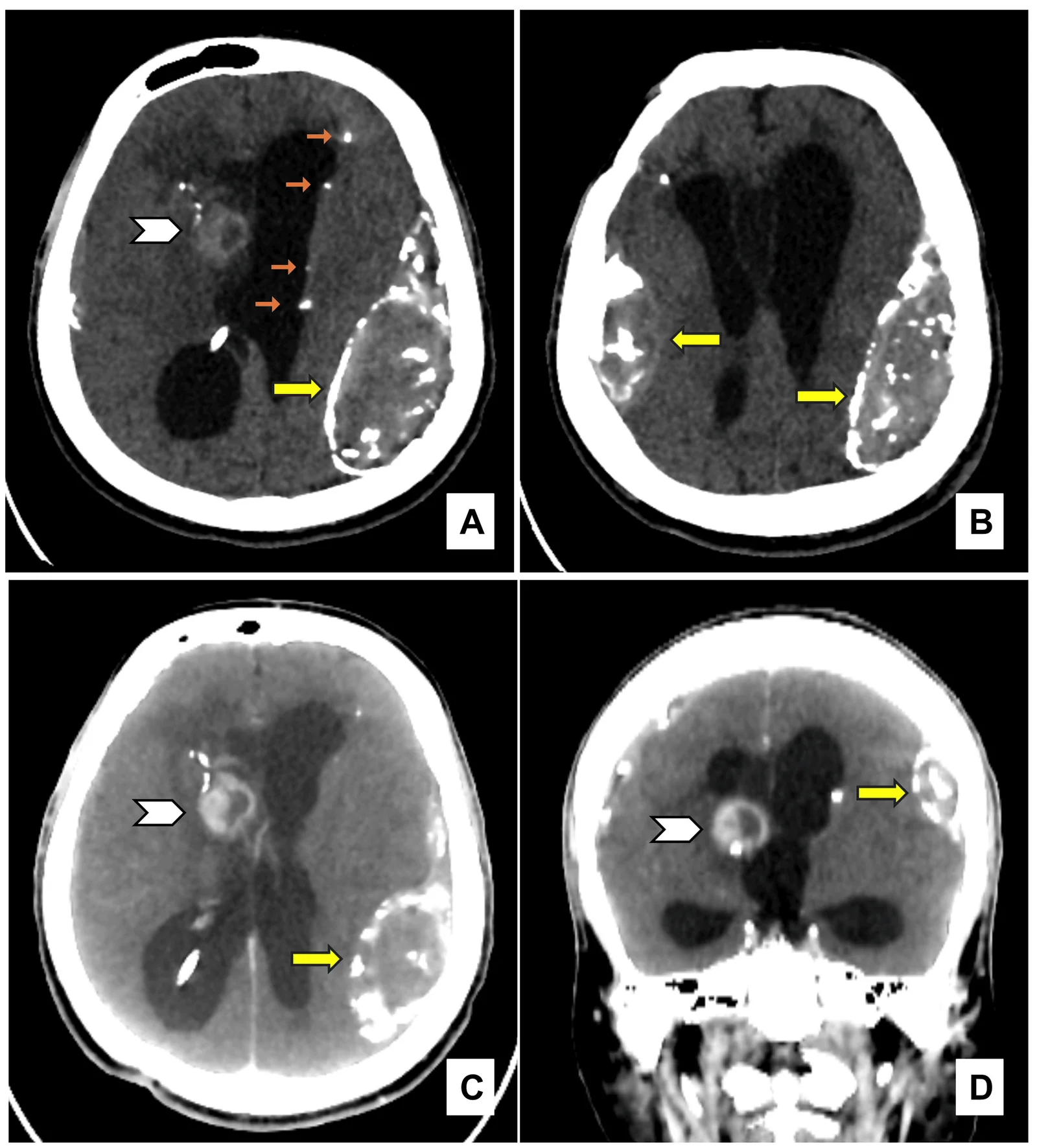
CT imaging of the intraventricular tumor and associated calcified lesions. (A, B) Non-contrast axial brain CT images (C) axial and coronal contrast-enhanced CT images (D) demonstrating an intraventricular tumor located near the foramen of Monro (arrowhead), associated with bilateral calcified subdural hematomas (yellow arrows) and calcified subependymal nodules (orange arrows).

Brain MRI performed after shunt revision demonstrated a heterogeneous enhancing right intraventricular mass centred at the foramen of Monro, with mixed solid and cystic components. Areas of signal void on susceptibility-weighted imaging (SWI) corresponded to calcified and hemorrhagic components (Figure 2). 

**Figure 2:**
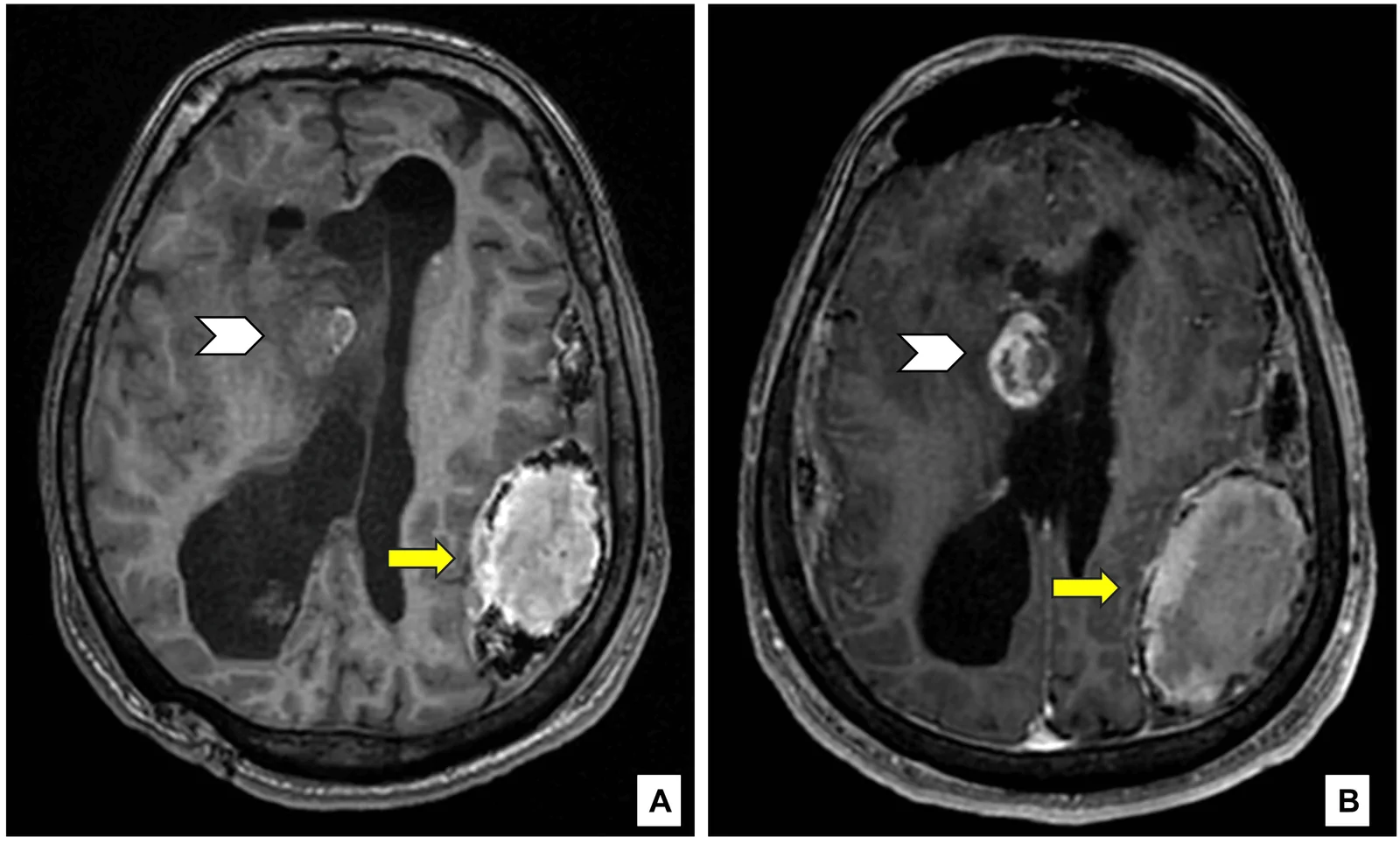
MRI features of the intraventricular tumor. (A) Non-contrast axial T1-weighted MRI and (B) post-contrast axial T1-weighted MRI demonstrating an intraventricular tumor located near the foramen of Monro, relatively well circumscribed with regular margins and heterogeneous enhancement (arrowhead) and hydrocephalus, associated to calcified subdural hematoma (yellow arrow).

The lesion exerted significant mass effect with associated subfalcine herniation, and tetraventricular hydrocephalus persisted. Additional findings included multiple subependymal nodules and cortical tubers appearing as fluid-attenuated inversion recovery (FLAIR) hyperintense lesions, which were identified only on MRI (Figure 3), as well as bilateral chronic calcified subdural hematomas (Figure 4).

**Figure 3:**
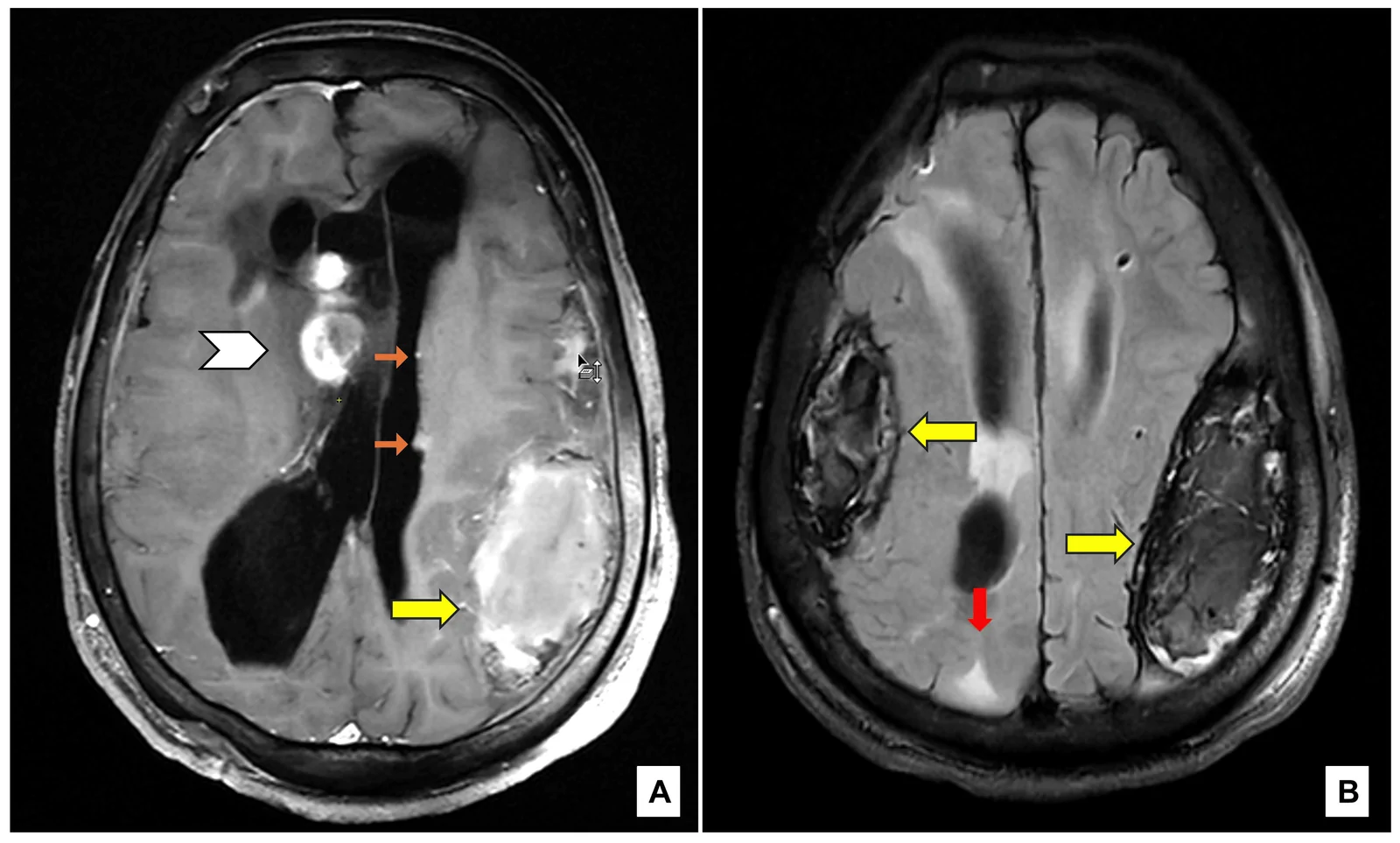
MRI demonstrating imaging features of tuberous sclerosis complex. (A) Post contrast axial T1-weighted and (B) axial T2-weighted FLAIR MRI sequences demonstrating an intraventricular tumor located near the foramen of Monro (arrowhead), associated with bilateral calcified subdural hematomas (yellow arrows), subependymal nodules (orange arrows), and cortical tubers (red arrow).

**Figure 4:**
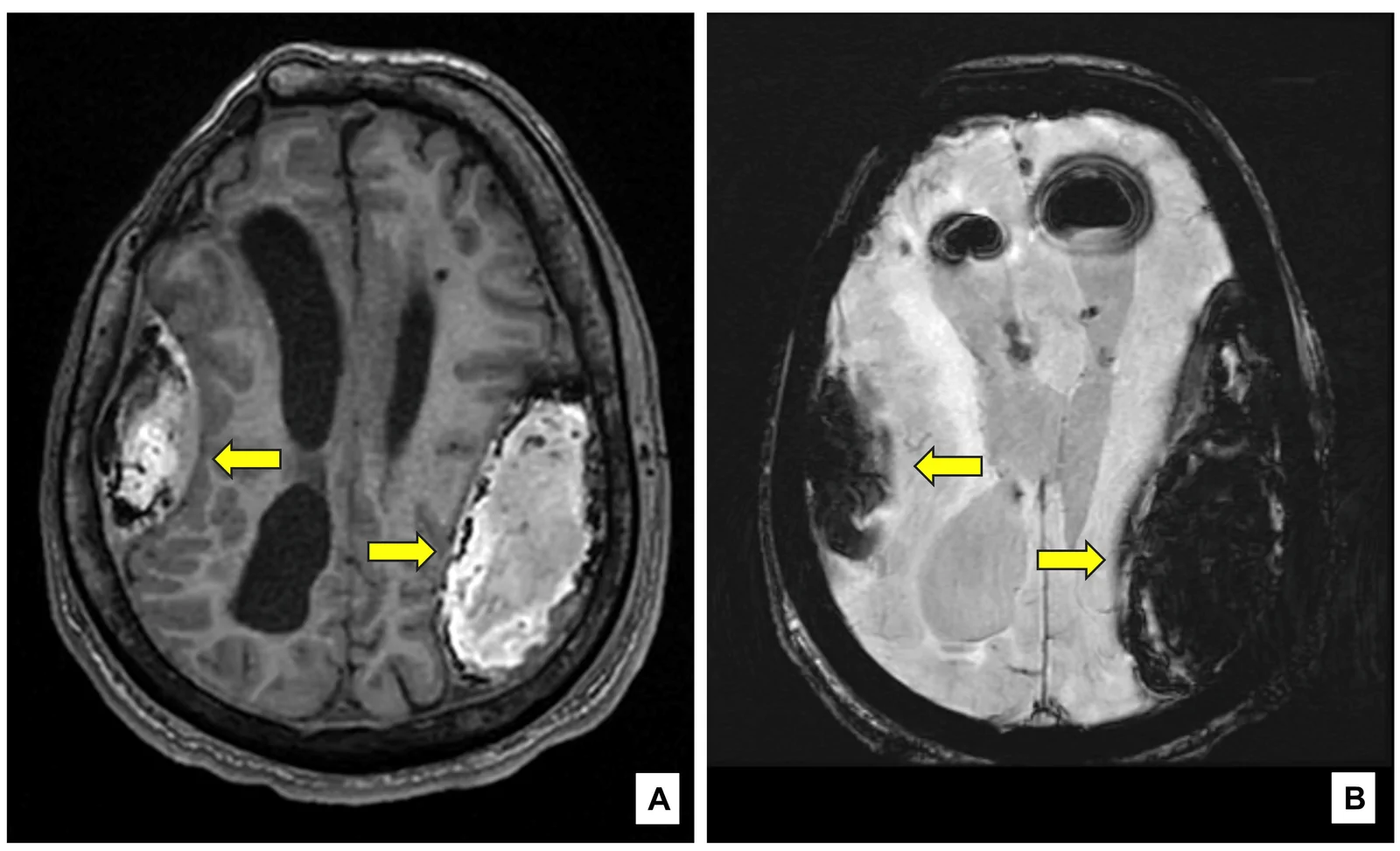
MRI features of bilateral calcified subdural hematomas. (A) Axial T1-weighted and (B) susceptibility-weighted imaging (SWI) MRI sequences showing bilateral calcified subdural hematomas (yellow arrows), appearing as large bilateral extra-axial lesions with characteristic signal features including T1 hyperintensity, and signal void on SWI with a surrounding hypointense rim.

These findings were suggestive of a subependymal giant cell astrocytoma associated with tuberous sclerosis complex. After clinical improvement following shunt revision, stereotactic biopsy of the lesion was performed. Histopathological examination confirmed the diagnosis of SEGA, as reported in the pathology report; however, detailed histological and immunohistochemical findings, including the Ki-67 proliferation index, were not available. Following shunt revision and biopsy, the patient's neurological symptoms improved, with resolution of the headaches and blurred vision. The patient remained neurologically stable during the 3-month follow-up period and was scheduled for surgical resection of the lesion. Renal and cardiac evaluations, spinal MRI, and genetic testing were recommended to further assess for systemic manifestations of TSC but were not performed because of financial constraints.

## Discussion

SEGA is a rare benign circumscribed astrocytic glioma classified as a World Health Organization grade 1 tumor [2]. It occurs almost exclusively in association with TSC and represents the most common intraventricular tumor observed in this phakomatosis. SEGA develops in approximately 5-20% of patients with TSC and typically arises during childhood or adolescence in the region of the foramen of Monro, predisposing patients to obstructive hydrocephalus [1,3].

According to the 2021 International Tuberous Sclerosis Complex Consensus criteria, the association of cortical tubers, subependymal nodules, hypomelanotic macules, and SEGA establishes a definite diagnosis of TSC. TSC is diagnosed based on a combination of clinical and genetic criteria, with SEGA representing one of the major diagnostic features [1].

Computed tomography (CT) is often the first-line imaging modality for the evaluation of SEGA and is particularly useful for detecting calcifications, hydrocephalus, and associated subependymal nodules. Typically, SEGA appears as a well-defined intraventricular mass located near the foramen of Monro, frequently demonstrating calcifications and contrast enhancement. Magnetic resonance imaging (MRI) remains the imaging modality of choice for lesion characterization and assessment of associated findings of TSC. On MRI, SEGAs usually demonstrate heterogeneous signal intensity with frequent cystic components appearing hyperintense on T2-weighted images, intense post-contrast enhancement, and occasional hemorrhagic changes [3,5]. These imaging characteristics, including a well-circumscribed intraventricular mass near the foramen of Monro, calcifications, contrast enhancement, and possible cystic or hemorrhagic components, were all present in our case.

In this case, the patient’s history of a histopathologically confirmed intraventricular ependymoma initially suggested tumor recurrence. However, recurrent ependymomas tend to manifest as more diffusely infiltrative lesions on MRI, often with signal and enhancement patterns similar to those of the primary tumor [4]. In contrast, the presence of multiple subependymal nodules and cortical tubers in our patient provided distinctive imaging features suggestive of TSC and raised the possibility of an alternative diagnosis. Although the childhood ependymoma had been confirmed histopathologically at that time, the original histopathological material is no longer available for re-evaluation. Therefore, the initial diagnosis cannot be retrospectively reassessed. In light of the current diagnosis of SEGA associated with TSC, we raise the possibility that the childhood lesion may have represented a SEGA that was initially misdiagnosed as an ependymoma. Recent reports have described delayed diagnoses of SEGA [6], underscoring the diagnostic complexity of these lesions and the importance of systematically evaluating imaging features suggestive of TSC. A comparison between our case and previously reported cases is presented in Table 1. It should be noted that the reported cases in the literature mainly involve patients of similar age, predominantly adolescents [7-9] (except for those diagnosed later in adulthood)[6], presenting with an intraventricular mass located near the foramen of Monro and causing obstructive hydrocephalus. This consistency reinforces the typical clinical and radiological presentation of SEGA in the context of tuberous sclerosis complex.

**Table 1:** Comparative review of reported cases of subependymal giant cell astrocytoma associated with tuberous sclerosis complex. SEGA, subependymal giant cell astrocytoma; TSC, tuberous sclerosis complex; mTOR, mammalian target of rapamycin; VP, ventriculoperitoneal; EVD, external ventricular drainage.

Features	Current case	Alassiri et al. [7]	Barkiche S et al. [8]	Nakamura et al. [9]	Zaher Addeen et al. [6]
						Age	18 years	14 years	12 years	14 years	33 years
SEGA characteristics	Intraventricular SEGA mimicking recurrent ependymoma	Intraventricular SEGA extending into the third ventricle	Intraventricular SEGA near the foramen of Monro	Intraventricular SEGA near the foramen of Monro	Intraventricular SEGA
TSC features	Cortical tubers, calcified subependymal nodules, hypomelanotic macules	Subependymal nodule	Subependymal nodules, renal angiomyolipoma, no neurocutaneous stigmata	Confirmed TSC since neonatal period, cortical tubers, regressed cardiac rhabdomyoma	Angiofibromas, ungual fibromas, hypomelanotic macules, cortical tubers, subependymal nodules, renal lesions
Hydrocephalus	Present, obstructive	Present, obstructive	Present, obstructive	Present, obstructive despite mTOR therapy	Present, obstructive
Other findings	Bilateral chronic calcified subdural hematomas	Subdural hematoma occurred two months following surgical resection	TSC revealed by SEGA	Complex hydrocephalus mechanisms	Delayed adult presentation
Treatment and outcome	VP shunt revision followed by clinical improvement ; stereotactic biopsy, surgery recommended	EVD, surgical resection, and mTOR inhibitor therapy	EVD, stereotactic biopsy, and mTOR inhibitor, fatal outcome	mTOR inhibitors followed by surgical resection; favorable outcome	Microsurgical resection with EVD; complete recovery at 6 months

Our case is distinguished by the presence of bilateral chronic calcified subdural hematomas, as reported in the case cited in Table 1, where a subdural hematoma developed two months postoperatively during follow-up after tumor resection [7]. In our patient, these chronic calcified collections may have resulted from a previous postoperative hemorrhage related to the initial tumor resection; alternatively, given the history of ventriculoperitoneal shunting, shunt-related overdrainage may have contributed to their initial development, followed by organization and calcification over time. This radiological peculiarity underlines the importance of reviewing the patient’s neurosurgical history and recognizing long-term postoperative sequelae that may complicate imaging interpretation. Correct identification of SEGA is essential because its management differs from that of recurrent ependymoma.

In addition to surgical resection, mammalian target of rapamycin (mTOR) inhibitors, particularly everolimus, have demonstrated sustained efficacy in reducing SEGA volume, with tumor volume reductions of ≥30% and ≥50% reported during long-term treatment [10]. Accurate diagnosis determines therapeutic strategy (surgery versus medical treatment), prognosis, implementation of genetic testing (TSC1/TSC2), and long-term multisystem surveillance.

## Conclusions

In adolescents presenting with an intraventricular mass near the foramen of Monro and associated obstructive hydrocephalus, the identification of subependymal nodules and cortical tubers should raise suspicion for SEGA in the setting of TSC, even in patients with a documented previous diagnosis of ependymoma. In our case, the current imaging and histopathological findings supported the diagnosis of SEGA. Although the original histopathological material from the childhood lesion was unavailable for re-evaluation, the current diagnosis of SEGA in the setting of TSC raises the possibility that the previous lesion may have represented a SEGA rather than an ependymoma. This case underscores the importance of a thorough radiological assessment for associated manifestations of TSC when evaluating an intraventricular mass in this clinical context.
